# Protocol: a fast and simple *in situ* PCR method for localising gene expression in plant tissue

**DOI:** 10.1186/1746-4811-10-29

**Published:** 2014-09-18

**Authors:** Asmini Athman, Sandra K Tanz, Vanessa M Conn, Charlotte Jordans, Gwenda M Mayo, Weng W Ng, Rachel A Burton, Simon J Conn, Matthew Gilliham

**Affiliations:** 1 ARC Centre of Excellence in Plant Energy Biology, University of Adelaide, Glen Osmond, SA, Australia; 2 ARC Centre of Excellence in Plant Energy Biology, The University of Western Australia, Perth, WA, Australia; 3 Waite Research Institute & School of Agriculture, Food and Wine, University of Adelaide, PMB1, Glen Osmond, SA 5064, Australia; 4 Adelaide Microscopy Waite Facility, University of Adelaide, Glen Osmond, SA, Australia; 5 ARC Centre of Excellence in Plant Cell Walls, University of Adelaide, Glen Osmond, SA, Australia; 6 Centre for Cancer Biology, Division Immunology, Level 3, Frome Road, Adelaide, SA, Australia

**Keywords:** *In situ* PCR, RT-PCR, Cell-specific localisation, Plant tissue, Immunohistochemistry

## Abstract

**Background:**

An important step in characterising the function of a gene is identifying the cells in which it is expressed. Traditional methods to determine this include *in situ* hybridisation, gene promoter-reporter fusions or cell isolation/purification techniques followed by quantitative PCR. These methods, although frequently used, can have limitations including their time-consuming nature, limited specificity, reliance upon well-annotated promoters, high cost, and the need for specialized equipment. *In situ* PCR is a relatively simple and rapid method that involves the amplification of specific mRNA directly within plant tissue whilst incorporating labelled nucleotides that are subsequently detected by immunohistochemistry. Another notable advantage of this technique is that it can be used on plants that are not easily genetically transformed.

**Results:**

An optimised workflow for in-tube and on-slide *in situ* PCR is presented that has been evaluated using multiple plant species and tissue types. The protocol includes optimised methods for: (i) fixing, embedding, and sectioning of plant tissue; (ii) DNase treatment; (iii) *in situ* RT-PCR with the incorporation of DIG-labelled nucleotides; (iv) signal detection using colourimetric alkaline phosphatase substrates; and (v) mounting and microscopy. We also provide advice on troubleshooting and the limitations of using fluorescence as an alternative detection method. Using our protocol, reliable results can be obtained within two days from harvesting plant material. This method requires limited specialized equipment and can be adopted by any laboratory with a vibratome (vibrating blade microtome), a standard thermocycler, and a microscope. We show that the technique can be used to localise gene expression with cell-specific resolution.

**Conclusions:**

The *in situ* PCR method presented here is highly sensitive and specific. It reliably identifies the cellular expression pattern of even highly homologous and low abundance transcripts within target tissues, and can be completed within two days of harvesting tissue. As such, it has considerable advantages over other methods, especially in terms of time and cost. We recommend its adoption as the standard laboratory technique of choice for demonstrating the cellular expression pattern of a gene of interest.

## Introduction

The function of only a small fraction of genes from any species has been experimentally verified. For the majority of plant genes, their function are either unknown or have been predicted through homology with a known gene from another species [1]. With the advent of next generation sequencing, this disparity between annotated genes and those that are functionally characterized is likely to increase.

An important step toward understanding the role of a gene product is to identify the tissue and cell type within which it is expressed. However, transcript abundance is predominantly analysed using whole plants or organs. Despite the fact that few transcripts are expressed exclusively within a single cell type, many genes are preferentially expressed in only a few cell types [2]. Analysing the transcript abundance of such genes in whole plants or organs may be misleading and will reflect the net expression for those particular genes in all cells rather than in the cell types in which they are actually expressed. This is of particular relevance for genes expressed predominantly in cell types that comprise only a small percentage of the total analysed tissue. Even if transcripts of genes of interest are highly abundant in one or few cell types the total number of the transcripts of interest may be diluted to below the detection limits within the whole tissue RNA pool. Such a phenomenon is not uncommon as there are a number of specific cell-types that appear to have a profound influence on the performance of the plant and act as ‘gatekeepers’ for a particular physiological process [3,4]. These cell-types contain proteins and signalling cascades that are primed for a particular physiological role so have unique transcriptional profiles. Such cell-types include, but are not limited to, guard cells (that undergo large turgor changes to control whole plant gas exchange), xylem parenchyma (which control net xylem content so have a major influence on long-distance nutrient and water transport), and pericycle (which initiate lateral root development so have a major role in root architecture). The transcriptional profiling of whole tissues can therefore be extremely misleading when ascribing a function to a gene and caution should be observed when interpreting expression data from tissue samples containing more than one cell-type. Awareness of such issues has led to the development of techniques that have allowed researchers to examine the expression of specific genes in specific cell types.

Common assays for detecting where transcripts are expressed can be classified in several ways (Table 1). Ordinarily they rely on one of the following: 1) the generation of transgenic plants with indicator proteins or nucleic acid/ribosomal binding proteins; 2) the isolation of single cells or single cell contents; 3) the hybridisation of RNA/cDNA directly in tissue; or 4) a combination of 1 and 2. These techniques can be classified into two further groups: A) those that first isolate RNA from a tissue or specific cell-type and then identify a transcript of interest in that RNA pool or, B) those that search directly for the transcript of interest in living or fixed plant tissue. We have summarised the advantages and disadvantages of these methodologies in Table 1.

**Table 1:** **Techniques available for *in planta *** **cell level expression analysis**

**Technique**	**Type**	**Comments**	**References**
Specific gene promoter: indicator protein fusions [i.e. beta-glucuronidase (GUS) or fluorescent proteins]	1, B	Long lead times of >2 months to get stably expressed genes, homozygous gene expression, sequences other than the promoter may control gene expression, no guarantee that promoter fragment chosen is correct, not suitable for plants that cannot be transformed.	[5]
Laser Capture Microdissection (LCM) and Single Cell Sampling (SiCSA)	2, A	Difficulty in isolating certain cell-types (e.g. vascular cells using SiCSA), long tissue prep (~2 weeks) for LCM, need for specialised equipment	[6,7]
*in situ* PCR	3, B	No specialised equipment required apart from vibratome, simple and fast method. No detailed protocol for plants until this manuscript, especially for agarose- embedded vibratome- sectioned in tube PCR. Can be performed on plants that are difficult to, or cannot be transformed. We do not recommend fluorescence detection of *in situ* PCR products due to interference with autofluorescence from plant tissue. As it is possible to do separate PCRs on adjoining tissue sections or replicate tissues from other plants we see no great advantage in multiplexing *in situ* PCR as it causes multiple complications to what is a robust and relatively simple technique. We do not recommend multiplexing *in situ* PCR due to the differential abundance of transcripts in the same cell and consequently the saturation of products, generation of non-specific products and problems with signal separation.	
*in situ* hybridisation (ISH)	3, B	High detection threshold (10–20 copies per cell for ISH vs 1–2 copies per cell for *in situ* PCR), need to design a specific probe that hybridises to RNA while *in situ* PCR uses the same primers as qPCR, much cheaper. The View RNA Assay (Affymetrix) enables automated multiple transcript detection using fluorescence by employing the principles of ISH to detect nucleic acid targets within specific cells/cell-types. Its use is limited to abundant transcripts and laboratories equipped with and/or experienced in FFPE (formalin-fixed paraffin embedded) and frozen tissue preparation.	[8]
Protoplasting of fluorescently labelled cells and single cell sorting (FACS)	4, A	Can assay multiple transcripts at the same time. Potential damage responses of tissue. Needs specialised sorting flow cytometer. Limited to analysis of cells that are fluorescently labelled. Cannot give detail of all cells in which a particular gene is expressed.	[9,10]
Nuclear sorting, INTACT (isolation of nuclei tagged in specific cell types) or ribosomal binding techniques	4, A	When combined with microarrays or RNAseq can assay multiple transcripts at the same time. Limited to analysis of cells that are fluorescently labelled. Cannot give detail of all cells in which a particular gene is expressed. Not easily replicated.	[11-13]
*In situ* RNA sequencing	3, B	Offers the prospect of obtaining whole transcriptomes and more from single cells in tissue sections. Not yet optimised in any tissue. Has not been performed for plant tissue.	[14]

Many of these methods have long lead times as they require the generation of transgenic plants so in these instances can be unsuitable for species that cannot be transformed or for the analysis of multiple genes. Also, many of these methods are difficult to adopt in a laboratory not set up for such techniques without a significant outlay as they utilize expensive and specialized equipment and expertise. Of the non-transgenic techniques, *in situ* hybridization and *in situ* PCR are methods that allow gene expression analysis at a single cell resolution. Two disadvantages of *in situ* hybridization revolve around sensitivity. Firstly, low-copy mRNAs often result in a false negative signal and secondly, highly homologous members of the same gene family are difficult to distinguish. Both of these issues are addressed with *in situ* PCR because a specific region of the gene transcript is amplified during multiple cycles of the PCR resulting in a highly sensitive technique.

*In situ* PCR is a semi-quantitative method that has been used to detect target RNA using colourimetry or fluorescence. Therefore it is useful to detect in which cell types genes are expressed. To precisely quantitate transcript levels in tissues or single cells, *in situ* PCR should be used in conjunction with a quantitative transcript detection technique (such as Single cell sampling and analysis (SiCSA) in combination with RNASeq, qPCR, or microarrays (Table 1).

**Figure 1:**
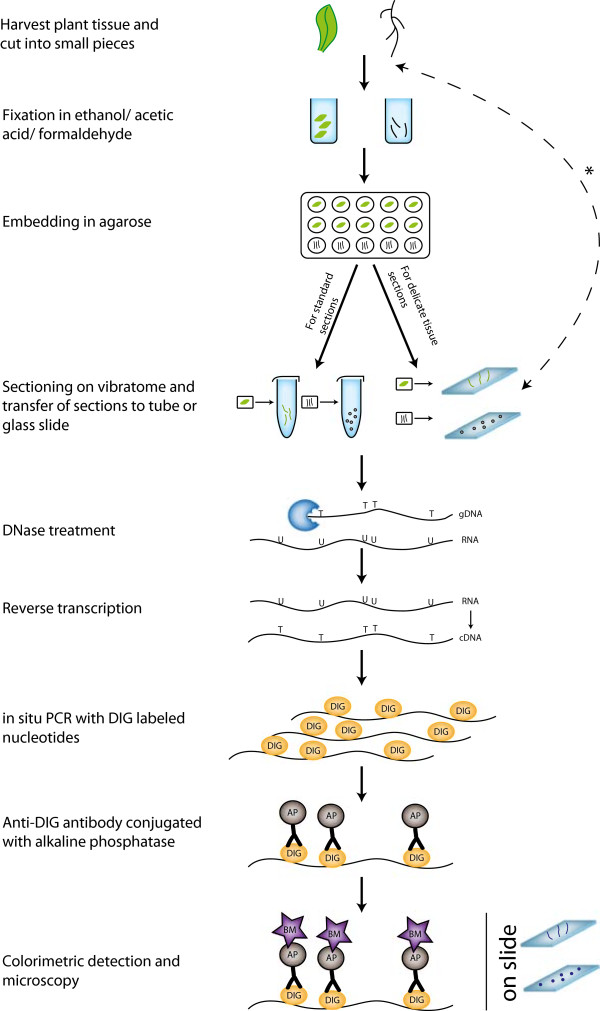
**Schematic representation of the *in situ *PCR pipeline.** Plant tissue is fixed in an ethanol/acetic acid/formaldehyde solution, followed by embedding in agarose and sectioning. Sections are collected into a microfuge tube in which DNase treatment, reverse transcription and *in situ* PCR are carried out using a thermocycler. During *in situ* PCR, DIG labeled nucleotides are incorporated into the PCR products. An anti-DIG antibody conjugated with alkaline phosphatase and an alkaline phosphatase substrate are used for the detection of the DIG labeled PCR products. These are visualized under a microscope. Thin fragile sections are placed onto a glass slide after sectioning, while *non-embedded, non-sectioned samples (i.e. epidermal peels) are placed directly onto slides for all processes from fixation to the final PCR step (dotted arrow).

*In situ* PCR has been described and performed extensively in animals and a detailed protocol has been made available [15]. Reports of *in situ* PCR on plant material either include lengthy tissue preparations, thermal cycling of slide-mounted specimens, or a detailed protocol was not supplied [16-20]. Here, we have further developed the *in situ* PCR technique, allowing improved resolution of transcript localisation. We also include detailed recommendations for the implementation of this technique in a variety of plant tissues to improve its utility (Figure 1). This includes both an in-tube protocol, which uses a standard thermocycler block for routine samples and an on-slide methodology, which uses a slide thermal cycler for use with fragile tissues. Although this alternative is not necessary for most applications of *in situ* PCR, it has advantages for very thin sections (<15 μm), or for delicate dissected tissues such as epidermal peels. We have used this method in published articles for the localisation of transcripts in wheat, barley and soybean [21-24], but due to the constraints of most journals have not had the opportunity to publish the methodology in the detail required for others to follow. Here, to outline the technique we include a full protocol and we show the preferential localisation of transcripts in various plant tissues and cell types, including guard cells, mesophyll, xylem parenchyma, pericycle, and different reproductive tissues.

## Protocol

### Overview

**Figure 2:**
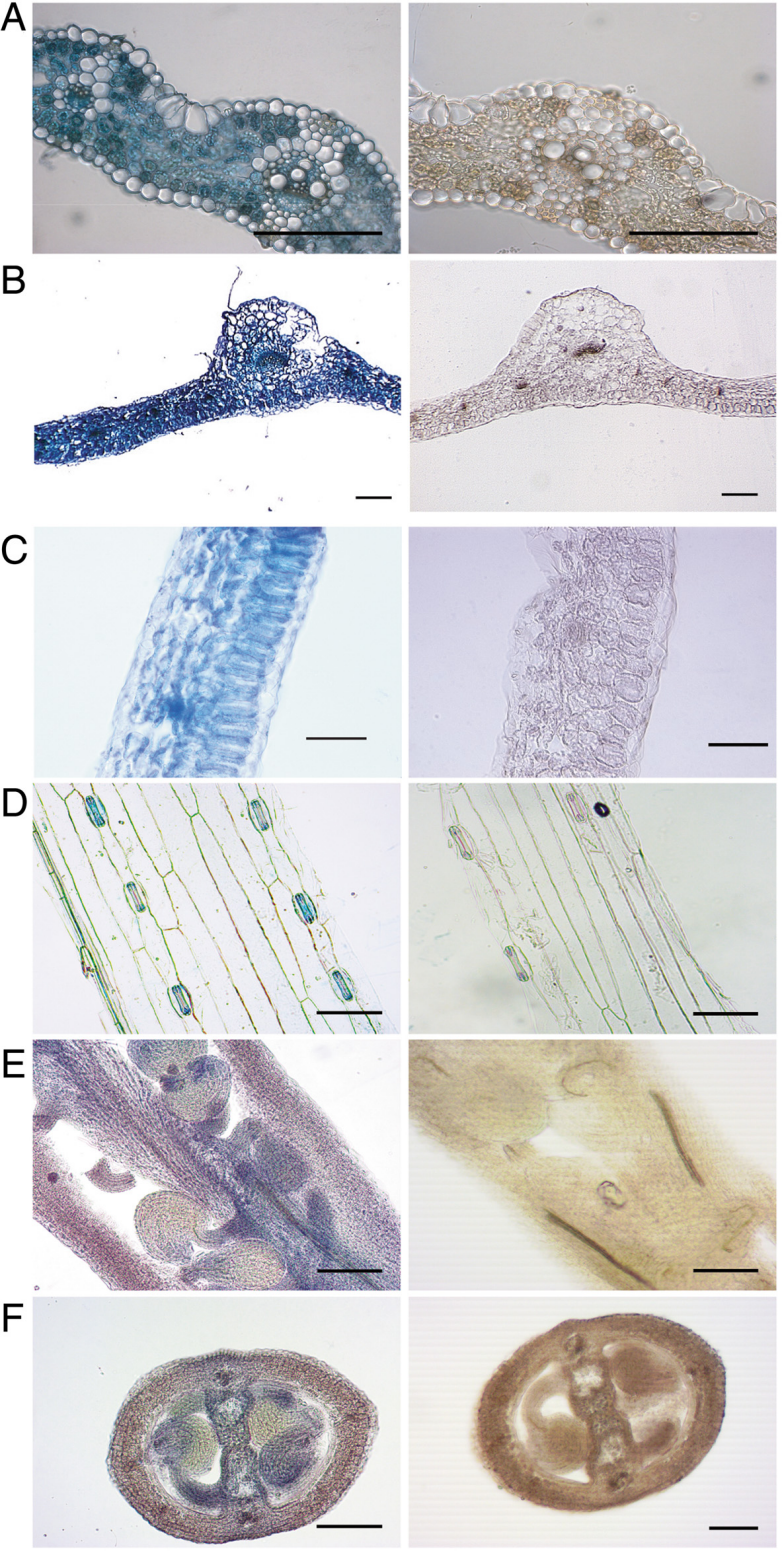
***In situ *PCR for various transcripts in barley and Arabidopsis.** Blue staining demonstrates presence of cDNA (left panel) while a brown colour indicates absence of amplified target cDNA within cells (right panel). **(A, left)** 18S ribosomal RNA positive control to show staining in all cell-types of barley leaf, **(B, left)** 18S ribosomal RNA positive control to show staining in all cell-types of Arabidopsis leaf, **(C, left)***AtCAX1* expression predominantly in the mesophyll of Arabidopsis leaf, **(D, left)** guard cell staining of a barley Aluminum-activated malate transporter *(ALMT)* (*SL1251*) from an *in situ* PCR performed on epidermal peels. **(E and F, left)** detection of an Arabidopsis *ALMT14* in the septum **(E)** and embryo **(F)** of developing flowers/siliques. The same primers and conditions were used for the corresponding negative controls (A-F, right panels, Table 6). Scale bars represent 100 μm.

To preserve the morphology of the plant tissue and to anchor the transcripts to their cellular origin, freshly harvested plant tissue is immediately fixed in an ethanol/acetic acid/formaldehyde solution with the penetration of the fixative enhanced through vacuum infiltration (Figure 1). The plant tissue is then embedded in agarose for subsequent sectioning on a vibratome (Figure 1). Sections are collected into a microfuge tube or placed on a slide. DNase treatment, reverse transcription and *in situ* PCR can all be carried out in the microfuge tube using a standard thermal cycler or on the slide using an *in situ* block. DNase treatment is carried out to remove genomic DNA (Figure 1), which would otherwise lead to erroneous detection of signal. The DNase-treated RNA in the tissue is converted to cDNA by reverse transcription (Figure 1). The resulting cDNA is amplified by standard gene-specific PCR integrating Digoxigenin (DIG) labeled nucleotides (Figure 1). The sections are incubated with an anti-DIG antibody conjugated to alkaline phosphatase, which binds to the DIG-labeled PCR products (Figure 1). Adding specific substrates for alkaline phosphatase allows the colourimetric detection of the DIG-labeled PCR products, delimited to the site of RNA synthesis. At this point, if the protocol was carried out in a microfuge tube, the sections are transferred onto a microscope slide (Figure 1). After mounting the slides, the processed tissue sections can be visualized by bright field microscopy (Figure 2).

### Plant growth and tissue sampling

The protocol described here concentrates on the amplification of RNA/cDNA from leaf and root tissues across a range of plant species (Table 2). By optimizing various aspects of the conditions, this method can be adapted easily to tissue types other than leaf or root (Table 2). The plant-growth method is inconsequential for the success of this technique, but we recommend hydroponics for root tissue samples to simplify harvesting of tissue [25]. Roots of plants grown in soil or agar should be immersed repeatedly in sterile water using a soft paintbrush to remove particulate matter while minimising damage to the roots.

**Table 2:** **Various alkaline phosphatase (AP) substrates used with this *in situ *** **PCR protocol on different plant species and tissues**

**Plant species**	**Tissue**	**AP substrate**
*Arabidopsis thaliana*	Leaf	BM Purple
		Elf97
		Vector Blue
*Arabidopsis thaliana*	Flower	BM Purple
*Cleome gynandra*	Leaf	BM purple
*Cleome hassleriana*	Leaf	BM purple
*Glycine max*	Root	BM purple
*Hordeum vulgare*	Leaf	BM purple
		Elf97
*Hordeum vulgare*	Epidermal peel	BM Purple
		Elf97^++^
*Oryza sativa*	Leaf	BM Purple
		Vector Blue
*Oryza sativa*	Root	BM Purple
*Solanum lycopersicum*	Leaf	Vector Blue
*Triticum aestivum*	Root	BM Purple
*Triticum durum*	Leaf	Elf97
*Triticum durum*	Root	BM Purple
		NBT/BCIP^X^
		Vector Blue^++^
*Vitis vinifera*	Root	Vector Blue
*Vitis vinifera*	Leaf	BM Purple
*Zea mays*	Leaf	BM Purple

Most AP substrates were used successfully for specific detection of the transcript in question, with some exceptions as indicated. ^++^indicates detection with significant background staining. ^X^indicates no detectable signal.

Prior to performing *in situ* PCR, it is important to consider the time of day that the tissue will be harvested, the growth stage of the plant, and the tissue type, as these factors may influence gene expression. As a general rule, we sampled at relative midday with respect to the photoperiod. For leaves, we use young and undamaged leaves, harvesting material with the midrib intact as this provides extra stability to the sections. Excising the leaf tip, outer margins and base (>2 mm from petiole) simplifies vibratome sectioning, handling and visualization of sections. We generally avoid older root sections with a large number of lateral root hairs as the root hairs tend to stick to the blade during vibratome sectioning, and this may result in whole root samples being pulled out of the agarose block. The time taken to prepare the material for fixation should be kept to a minimum as RNA may begin to degrade and tissue begins to wilt. In our experience we can prepare tissue in under 1 minute. This will not avoid very rapid changes in expression caused by handling; such changes are unavoidable in most applications. The number of leaves or roots to be harvested depends on the number of transcripts to be tested by *in situ* RT-PCR, with at least 3 reproducible biological replicates per tissue per condition being required to confirm expression patterns – this means that more than three tissue replicates will need to be harvested and fixed to account for damage or loss of tissue etc.

### Tissue preparation

The method of tissue preparation used in this protocol involves formaldehyde/acetic acid/ethanol fixation and agarose-embedding, a fast and simple method that preserves the morphology of the plant tissue and keeps the RNA molecules intact [26]. Formaldehyde is a small and highly permeable molecule [27], which accurately preserves cellular morphology, condensing fixation times of most samples to two hours. It is reported that fixation in excess of four hours for leaves and 12 hours for roots in formaldehyde can result in excessive cross-linking of proteins to nucleic acids [28,29], which can interfere with the subsequent reverse transcription resulting in no detectable staining or in significant background staining of the sample.

Following fixation, the tissue should be embedded in molten agarose and allowed to cool for at least 1.5 hours prior to sectioning. This is significantly shorter than the time required for paraffin embedding (2–7 days). The RNA in the agarose-embedded tissue is stable for at least three weeks and can be detected on plant tissue sections cut as thin as 20 μm using a vibratome. Better image clarity is observed with thinner sections but these are more easily damaged than thicker sections during the wash steps. We recommend 50 μm thick sections for roots and 60–70 μm sections for leaves. While the sections may not be as thin as those that can be cut from frozen or paraffin-embedded tissues (4–15 μm), cell morphology is well preserved and transcripts are easily detected following *in situ* PCR in tissue sections of up to 75 μm thick. Furthermore, the absence of the pre- and post-treatment steps that are required with paraffin embedding and cryosectioning, make agarose embedding a technique suitable for any standard laboratory without the need for specialized equipment.

Generally, we embed three leaf samples or eight root samples per agarose block. The sectioning of one of these agarose blocks should yield enough tissue sections to perform *in situ* PCRs on at least three transcripts. From our experience, to be confident in the localisation of a transcript we suggest that at least ten tissue sections are visualised per transcript per biological replicate to check that the expression patterns are consistent across tissues in at least three biological replicates. To account for loss or damage to samples during processing we recommend starting with three times the amount of sections during vibratome sectioning and at least one additional biological replicate (so examine tissues from four plants). During sectioning the tissue usually separates from the agarose, so the number of sections can be counted onto the slide or tube for processing.

In some cases (such as with epidermal peels or the seed aleurone), tissue can be dissected from the plant without the need for sectioning on a vibratome. The processing of these non-embedded tissues should be carried out entirely on glass slides from fixation through to the final PCR and staining steps. In addition, if thin (<50 μm) or particularly fragile sections are needed for PCR, then it is again best to avoid in-tube processing of these samples; instead, these tissues are best processed on slides. To do this, when sections are taken post-fixation and embedding, they should be collected directly onto the slide for the DNAse treatment step and through to the final PCR and staining steps. These variations in the sample processing are highlighted in the protocol below.

### Primer design

Primers should be designed to the cDNA sequence of a plant species. To test if the primer sequences align to genomic DNA (gDNA), the primer sequence can be compared against available gDNA sequences using BLAST (http://blast.ncbi.nlm.nih.gov/). The primers should amplify a 150–300 bp product, since this size product simplifies amplification within the cellular matrix and provides sufficient DIG incorporation (and thus signal) per amplicon. Oligonucleotides should be 18–24 bases long, have an annealing temperature of 55°C and if possible, span an exon-exon junction to prevent the amplification of gDNA. If the latter is not possible, the primers should be designed to span an intron sequence (i.e. the two primers are on two different exons) so that possible amplification from gDNA is eliminated by using a short extension time during the *in situ* PCR. The following standard primer design features should also be observed: they should amplify a single product; contain no internal secondary structure (hairpin, palindromes, self-dimers, repeats and runs in excess of four bases); contain 40–60% G/C; and, should not be self-complementary to avoid primer dimer formation. To assist with these guidelines, free online primer design and primer analysis tools can be used, such as Primer3 (http://simgene.com/Primer3) and NetPrimer (http://www.premierbiosoft.com/netprimer/).

### Optimization of PCR conditions

Before conducting *in situ* PCR on any plant tissue, the amplification conditions must be optimised in solution, such as the concentration of magnesium and dNTPs, the type of polymerase used, the annealing temperature, extension time and cycle number. Optimised parameters invariably transfer successfully to the *in situ* PCR protocol. To determine the number of cycles that should be used for the *in situ* PCR, it is critical to choose the minimum cycle number that gives an identifiable product to maximise the chance of differentiating abundance between cell types, albeit semi-quantitatively, for the gene of interest. The cycle number used for *in situ* PCRs may be different for each gene to be tested. When choosing a cycle number for the *in situ* PCR, it needs to be considered that the PCRs are not likely to be as efficient for cDNA contained within plant tissue as compared to cDNA in solution, and the incorporation of DIG-dUTP during the *in situ* PCR may have an inhibitory effect on the DNA polymerase. Considering the above points, adding 1–2 cycles to the cycle number obtained from the in-solution PCR is recommended when conducting the *in situ* PCR.

### RT-PCR

Since *in situ* PCR is primarily used for detecting a particular mRNA, an *in situ* reverse transcription (RT) step precedes the *in situ* PCR. During the RT, mRNAs are converted to cDNAs, either by a random primed or a specific primed RT reaction. Random primed RT reactions use random hexamers that are six bases long and convert all RNA molecules (mRNAs, rRNA and ncRNAs) into cDNA. We generally use the specific primed RT reaction, where a specific primer designed to the 3′ end of the transcript of interest is used to reverse transcribe only the required transcript from the mRNA. A fragment of this transcript is then amplified in the following PCR using either a pair of nested gene-specific primers or the RT-primer plus a gene-specific primer 5′ of this. For the design of the RT primer, the standard primer design guidelines apply as described above under ‘Primer design’.

### Amplicon labeling and detection

Amplicons can be labeled directly with a fluorescent label, which allows their subsequent detection by microscopy, or indirectly using either digoxigenin (DIG) or biotin. DIG-labeled PCR products can be detected using enzyme-based colour detection systems such as alkaline-phosphatase (AP) or peroxidase, using an anti-DIG-enzyme conjugate that binds to the DIG label on the amplicon. Biotin-labeled amplicons can be recognized using a streptavidin-enzyme conjugate for colourimetric detection in a similar fashion. We had most success with the colourimetric detection of an anti-DIG-AP conjugate that recognizes DIG-labeled-dUTP incorporated into the amplicon during the *in situ* PCR. We have tested different AP substrates with varying success for different plant tissues and species (Table 2). This table includes fluorescent substrates but we found these problematic due to significant detection of autofluorescence from the tissue interfering with our substrate fluorescence (see Results and discussion).

### Validation and controls

To validate the expression pattern of a gene, we routinely perform *in situ* PCR on replicate tissues from four different plants. In addition, as mentioned above, multiple technical replicates (~10) are useful per biological replicate to validate the site of amplification and detection. A negative control that tests for the presence of contaminating gDNA should be included alongside each transcript analyzed by *in situ* PCR. This consists of tissue samples, prepared as for the test samples but with the omission of the reverse transcriptase enzyme during the RT step. A positive control should also be included for each tissue sample tested. This should be a gene that is uniformly expressed in all cells; if the effect of a treatment is being tested in your experiment, this gene should remain stably expressed across different treatments. The ribosomal 18S transcript, which is expressed across the majority of plant species and is ubiquitously expressed in all cells, is recommended.

## Materials

### Reagents

•RNase Zap (Sigma, cat. no. R2020).

•Fixative (see REAGENT SETUP).

•Ethanol (Chem-supply, cat no. 64-17-5, 100% Analytical Reagent).

•Acetic acid (Southern Cross Science, cat no. AA009-2.5LP).

•Formaldehyde (Sigma, cat. no. 252549-4 L).

•Wash Buffer 1 (see REAGENT SETUP).

•1× PBS (see REAGENT SETUP).

•Agarose (Bioline, cat. no. BIO-41025) (see REAGENT SETUP).

•Ultra-low gelling temperature agarose (Sigma, cat. no. A2576) (see REAGENT SETUP).

•1 L sterile water (ice-cold).

•40 U μL^−^1 RNaseOUT (Invitrogen, cat. no. 10777019).

•10× Turbo DNA-free Buffer from Turbo DNA-free kit (Ambion, cat. no. AM1907). Only use the buffer TURBO DNaseI did not work well on plant sections.

•RNase-Free DNase Set (1500 U, lyophilised; Qiagen, cat. no. 79254) (see REAGENT SETUP).

•0.5 mM EDTA, pH 8.0 (Chem-Supply, cat no. 6381-92-6) (see REAGENT SETUP).

•dNTPs (10 mM each; Bioline, cat. no. BIO-39025).

•Thermoscript RT kit (Invitrogen, cat. no. 11146–024).

•RT reaction solution (see REAGENT SETUP).

•HiFiPhusion polymerase (Thermo Scientific, 2 U μL^−1^, cat. no. F-530S).

•Digoxigenin-11-dUTP, alkali stable (DIG-11-dUTP, 1 mM; Roche, cat. no. 11093088910).

•Gene-specific forward and reverse primers.

•PCR reaction solution (see REAGENT SETUP).

•1× Block solution (see REAGENT SETUP).

•Anti-Digoxigenin-AP, Fab fragments (Anti-DIG antibody; Roche, cat. no. 11093274910) (see REAGENT SETUP).

•Wash Buffer 2 (see REAGENT SETUP).

•AP substrates: BM Purple AP Substrate (Roche, cat. no. 11442074001) or Vector Blue Substrate Kit (Abacus ALS, cat. no. VESK5300) or NBT-BCIP solution (Sigma-Aldrich, cat. no 72091) or ELF97 Endogenous Phosphatase Detection Kit (Life Technologies, cat. no. E-6601).

•40% glycerol (VWR, cat. no. 24397.296) (see REAGENT SETUP).

•Clear nail varnish.

### Equipment

•Micro scissors (Wescott spring scissors; Proscitech, cat. no. TS1084).

•Soft paintbrushes

•Tweezers (ProSciTech, cat no. T045-212).

•Sterile scalpel (ProSciTech, cat.no. METP2325-10-CE).

•Sterile 150 mm petri dishes (Sarstedt, cat.no. 82.1184.001).

•2 mL microfuge tubes (VWR, cat. no. 211–2120).

•Vacuum infiltrator (EYELA Aspirator, cat. no. A-3S).

•3 MM Whatmann filter paper (diameter 90 mm; VWR, cat. no. WHAT1001-090). Sterilize by autoclaving.

•Sterile 12-well plates (Adelab, cat. no. CNG3513).

•0.2 mL PCR tubes (Adelab, cat. no. AXYPCR-02-C).

•Vibratome Leica VT1200 with magnetic specimen plate, buffer tray and ice bath (see EQUIPMENT SETUP).

•Superglue (Selleys Quick Fix).

•Double-edge blade (ProSciTech, cat. no. L056).

•Single-edge blade (ProSciTech, cat. no. L055C).

•Thermal cycler (Geneworks, cat. no. GS00001).

•*In-situ* block (Geneworks, cat. no. GSB0SITU).

•Microscope slides (ProSciTech, cat. no. G300B) or StarfrostSilane-prep slides (Sigma, cat. no. S4651-72EA).

•Frame-seal slide chambers (BioRad, cat. no. SLF0601).

•Coverslips (ProSciTech, cat.no. GCC2222).

•Glass pipette.

•For brightfield imaging: Leica, AS LMD microscope.

•For fluorescence detection: Zeiss Axiophot microscope: DAPI, BP 365/12, FT 395, LP 397.

•OR, Nikon A1R laser scanning confocal microscope: UV excitation using DAPI laser line (405 nm ex).

### Reagent setup

**Fixative** 63% Ethanol, 5% acetic acid, 2% formaldehyde. Prepare fresh and use within 3 h. Keep on ice.

Caution

Hazardous chemicals should be handled in a fume cupboard using appropriate personal protective equipment.

**Wash Buffer 1** 63% Ethanol, 5% glacial acetic acid. Prepare fresh and keep on ice.

Caution

Hazardous chemicals should be handled in a fume hood using appropriate personal protective equipment

**10× PBS stock solution, pH 7.5** 0.1 M Na_2_HPO_4_, 1.3 M NaCl, pH 7.5. Dissolve 14.19 g Na_2_HPO_4_ in 800 mL of sterile water. Adjust to pH 7.5 by adding 37% HCL. Add 75.9 g of NaCl and add sterile water to a total volume of 1 L. This solution can be kept for 1 year at room temperature.

**1× PBS, pH 7.5** Dilute the stock 10× PBS at 1:10 ratio to give a final concentration of 0.01 M Na_2_HPO_4_ and 0.13 M NaCl. This solution can be kept up for 1 year at room temperature.

**5**% **Agarose** or **5% Ultra-low gelling agarose** Dissolve 2.5 Agarose (leaves) or ultra-low gelling agarose (roots) in 50 mL 1× PBS. Can be kept at 4°C for 6 months.

Critical step

Ultra-low gelling agarose is recommended for embedding root tissue, while regular agarose works best on leaves. On the day of use, melt the agarose by heating in a water bath in a microwave oven under medium power and keep in a water bath/incubator at a temperature higher than the gelling temperature. We incubate at 50°C for 5% regular agarose and 37°C for 5% ultra-low gelling agarose.

**RNase-Free DNase Set** (lyophilised) Add 1.5 mL of RNase-free water (from kit) to lyophilized DNase I to give final concentration of 1 U μL^−1^.

Critical step

Buffer of this kit is optimized for on-column DNA digestion; do not use it. It is recommend to use 10× Turbo DNA-free Buffer from Turbo DNA-free kit (Ambion) (see REAGENTS).

**0.5 mM EDTA, pH 8.0** Add 18.6 g EDTA to ~80 mL of water. Adjust pH to 8.0 with NaOH. The disodium salt of EDTA will not go into solution until the pH is approximately 8.0. Add water to a total volume of 100 mL and sterilize by autoclaving.

**RT reaction solution** Use Thermoscript RT kit (see REAGENTS) and make up the first master mix according to the manufacturer’s instructions and the number of reactions. This initial master mix will contain reverse primer, dNTPs, water and tissue sections. An aliquot of the second master mix (Table 3) is added to the tube or slide containing the first master mix after denaturation of the first master mix.

**Table 3:** RT reaction solution

**Component**	**Stock conc.**	**Final conc./amount**	**1× (μL, in-tube)**	**1× (μL, on-slide)**
cDNA synthesis buffer (kit)	5×	1×	4	20
DTT (kit)	0.1 M	5 mM	1	5
RNaseOUT (kit)	40 U μL^−1^	40 U	1	1
Thermoscript RT (kit)	15 U μL^−1^	15 U	1	1

**PCR reaction solution** Use HiFi Phusion polymerase kit (see REAGENTS) and make up a master mix on ice according to the number of reactions (Table 4).

**Table 4:** PCR reaction solution

**Component**	**Stock conc.**	**Final conc./amount**	**1× (μL, in-tube)**	**1× (μL, on-slide)**
HiFi Phusion buffer	5×	1×	10	20
dNTPs	10 mM	0.2 mM	1	2
DIG-11-dUTP	1 mM	4 μM	0.2	0.4
HiFi Phusion polymerase	2 U μL^−1^	1 U	0.5	0.5
Sterile water			33.3	67.1

**10× Block stock solution** Mix 10 mg BSA in 1 mL 10x PBS. May be stored at −20°C for 1 year.

**1× Block solution** Just before use, dilute 10× Block stock solution 1:10 with sterile water to give 1× Block solution with a final concentration of 1% BSA in 1× PBS.

Critical step

Ensure you make enough 1× Block solution for blocking step and dilution of the Anti-DIG antibody. Need approximately 150 μL 1× Block solution per sample.

**Anti-Digoxigenin-AP Fab fragment** This is the Anti-DIG antibody conjugated with alkaline phosphatase (AP). Dilute Anti-DIG-AP Fab fragments 1:500 using 1× Block solution. For 10 samples prepare 550 μL: dilute 1.1 μL Anti-DIG-AP in 548.9 μL 1× Block.

**Wash Buffer 2** 0.1 M Tris-Cl, 0.15 M NaCl, pH 9.5. Can be kept at 4°C for 1 year.

**40% glycerol** Dilute 4 mL of 100% glycerol with water to give a final volume of 10 mL.

### Equipment setup

**Vibratome setup** The Leica VT1200 vibrating microtome comes with a magnetic specimen plate, buffer tray and ice bath. Assemble the vibratome according to the manufacturer’s instructions. Add sterile ice-cold water to the buffer tray. The water provides a flotation medium for the sections. To maintain the cold temperature, fill the integrated ice bath with crushed ice.

**Frame-seal slide chambers** If performing the *in-situ* PCR directly on slides the frame-seal should be adhered to the slide following the manufacturer’s instructions. Label all slides with species, gene name, whether negative or positive control, and the date.

**Thermal cyclers** If performing in-tube PCR, any standard thermal cycler is suitable. If performing reactions on slides, a thermal cycler with a block that can hold slides is necessary (see EQUIPMENT).

## Procedure

Critical step

General guidelines for working with RNA apply to avoid contamination with RNases. All solutions need to be RNase-free. Clean all surfaces and utensils with Ethanol and RNase Zap (see *Reagents*) or equivalent. Use filter tips or new sterile tips for RNA work only.

### Sample preparation and fixation

#### Timing ~3.5 h (leaves), ~20 h (roots), ~1.5 h (non-embedded, non-sectioned samples)

**1** Prepare fixative fresh and before harvesting plant material (see REAGENT SETUP). Place on ice.

Caution

Hazardous chemicals should be handled in a fume hood, and using appropriate personal protective equipment.

**For non-embedded, non-sectioned samples:** If the material to be tested is less than 50 μm thick (i.e. only 1–2 cell layers as for epidermal peels or aleurone layers), the agarose embedding and vibratome sectioning steps (Figure 1) are omitted from the protocol. All processes from fixation through to *in situ* PCR and staining are performed directly on a microscope slide.

**2** Aliquot 1.8 mL fresh fixative into 2 mL microfuge tubes (1 per species and tissue) and place on ice.

**For non-embedded, non-sectioned samples:** Aliquot 100 μL fresh fixative onto the rectangular frame seal slide chamber on a glass slide and keep on a cold flat surface.

**3** Harvest plant tissue using micro-scissors and place onto a sterile surface (i.e. petri dish). Using a sterile scalpel, cut leaves into 3 × 5 mm pieces and roots 5 mm long making sure each cut is perpendicular to the long axis of the tissue. For 10 *in situ* PCRs it is recommended to prepare 12 leaf pieces (4 agarose blocks with 3 samples each) and 32 root pieces (4 agarose blocks with 8 samples each).

**For non-embedded, non-sectioned samples:** The samples are placed directly onto the glass slides containing the fixative and incubated three times for 15 min on a cold flat surface, changing the fixative every time. Proceed to step 7 (washes).

Critical step

Samples should be limited in size to a maximum of 5 mm in width or length, as the fixative can only penetrate up to 5 mm through the tissue. Include the middle vein in leaf pieces as this assists in maintaining the integrity of the leaf sections during the procedure.

**4** Using a soft paintbrush, transfer leaf or root pieces immediately into 1.8 mL ice-cold fixative in 2 mL tubes. Carefully invert tubes three times and ensure samples are fully submerged and move unrestricted. This is done to ensure fixative is saturating and can easily access all surfaces of the samples. If the samples do not float freely, transfer some of the samples to a second tube with cold, fresh fixative.

Critical step

The time between harvesting the plant tissue and submerging it in fixative should be as short as possible.

**5** Vacuum infiltrate (400 mm Hg) twice for 1.5 min (2 mL tubes open lid). Between infiltrations, cap tubes and gently mix samples.

**6** Incubate leaf samples for 3–4 h in fixative on ice. Incubate roots for at least 12 h in fixative in the dark at 4°C. For leaf samples only: During the 3–4 h incubation time change the fixative every hour taking care not to damage the tissue. Keep tubes on ice in fume hood.

### Washes

#### Timing 1 h

**7** Wash three times for 10 min in 1.8 mL of ice-cold Wash Buffer 1 (see REAGENT SETUP). After last wash, remove as much of the wash buffer as possible.

**For non-embedded, non-sectioned samples**: Use 100 μL of Wash Buffer 1.

**8** Wash three times for 3 min in 1.8 mL of ice-cold 1x PBS (see REAGENT SETUP). After last wash, remove 600 μL of PBS and discard.

**For non-embedded, non-sectioned samples**: Wash three times in 100 μL of 1×PBS, add 85 μL sterile water and 2.5 μL of 40 U μL^−1^ RNaseOUT (=100 U) (see REAGENT SETUP) to each slide to give a final volume of 87.5 μL then proceed directly to step 29 (DNase treatment).

### Embedding

#### Timing ~1 h

**9** Place three sterile 3MM Whatman filter papers into the lid of a round petri dish (1 per sample).

Steps 10 and 11, process one sample at a time:

**10** Invert contents of a 2 mL tube over the filter paper in the petri dish lid. Use a soft paintbrush to gently transfer any remaining samples onto filter paper.

**11** In a sterile 12-well plate, half-fill a well with the molten 5% agarose (leaves) or ultra-low gelling agarose (roots) (see REAGENT SETUP). Immediately transfer tissue pieces into the molten agarose using a soft paintbrush. Ensure tissue pieces are fully submerged in the agarose. After orienting the tissue pieces, place 12-well plate on ice for agarose to solidify.

Critical step

Leaves: Place three leaf pieces in each well. Using two paintbrushes, orient the leaf pieces with flat faces parallel to each other and each epidermis perpendicular to the bottom of the plate (so the leaf pieces are stacked like the leaves of a book). Repeat at least four times in four different wells.

Roots: On filter paper, gently roll 8 root pieces parallel to each other to form a tight bundle. Using two paintbrushes or gloved hands transfer one bundle (8 root pieces) into an agarose-filled well, orienting the long axis (epidermis) perpendicular to the bottom of the plate. Repeat at least four times in four different wells.

**12** Seal plates using parafilm to prevent agarose drying out and store samples at 4°C for at least 3 h prior to sectioning to ensure the agarose is solidified thoroughly for sectioning on a vibratome. RNA should remain stable for at least three weeks.

PAUSE POINT.

### Sectioning

#### Timing ~3.5 h

**For thin fragile sections:** If sections thinner than 50 μm are desired from agarose embedded samples, we recommend proceeding on silanised glass slides [15] from this point to minimise tissue disturbance. Tissue sections are held in place between glass slide and plastic seal of frame-sealed chambers preventing the sections from coming in contact with the pipette tip, which would otherwise cause tissue damage.

**13** Prepare 0.2 mL PCR tubes and keep on ice during vibratome sectioning (1 tube per transcript to be tested, including controls):

**For both non-embedded, non-sectioned samples AND thin fragile sections:** Prepare frame-seal chambers on silane glass slides [15] and pipette water and RNaseOUT onto the slides according to ‘leaf tissue’ volumes below.

For leaf tissue, add 85 μL sterile water and 2.5 μL of 40 U μL^−1^ RNaseOUT (=100 U) (see REAGENT SETUP) to each tube to give a final volume of 87.5 μL. Leaf sections will be placed into this solution.

For root tissue, add 5 μL sterile water and 2.5 μL of 40 U μL^−1^ RNaseOUT (=100 U) (see REAGENT SETUP) to each tube. Four times 20 μL sterile water containing root sections will be added to this solution to give a final volume of 87.5 μL.

**14** Using a sterile scalpel, cut a square agarose block from a well in the 12-well plate, taking care not to cut the embedded tissue. Remove agarose edges. Use tweezers to lift the square agarose block onto a sterile petri dish. Alternatively, flip the 12-well plate upside down and then place the block on a petri dish. Using a sterile scalpel cut off excess agarose closer to the tissue to reduce the size of the block, taking care not to cut the embedded tissue.

**15** Place superglue onto the magnetic vibratome specimen plate slightly off centre (so agarose block can be orientated later). The amount should be enough to cover the base of the block. Immediately place agarose block onto superglue using tweezers or gloved hands.

**16** Place the specimen plate into its buffer tray, cover the tray with its lid and place on ice for at least 5 min for the superglue to dry.

**17** Fill the buffer tray with ice-cold sterile water until the agarose block is just covered. The cooled water provides a flotation medium for the sections.

**18** Place the buffer tray into the ice bath and fill the ice bath with crushed ice to maintain the cold temperature of the water.

**19** Assemble ice bath/buffer tray/specimen plate onto vibratome.

**20** Place a double-edge blade into the holder, tighten and turn into position for sectioning. The blade should sit just above the agarose block and be in contact with the water.

**21** If required, orient the agarose block by turning the specimen plate. Section leaves lengthwise along the leaf. This helps to keep the leaf sections intact and prevents leaf pieces to be pulled out of the agarose block.

**22** Before setting section thickness for cutting (raising the stage), move the blade back until ~0.5 cm behind the agarose block. Set thickness of sections for preliminary trimming at ~200 μm to cut away any excess agarose until the tissue is reached. Set velocity (0.4 mm sec^−1^) and amplitude (0.4 mm).

**23** Press ‘Run’ to cut section and again to stop. Remember to reverse blade before raising stage for next cut.

**24** Once the blade is in contact with the tissue, continue sectioning at 60–70 μm (leaves) or 50 μm (roots). Repeat 6–7 times. These first few sections will be damaged from processing and can be discarded. Collect the sections from the next 3 cuttings onto a microscope slide.

Critical step

Generally, the sections will split from the agarose and float into the cold water. Use a soft paintbrush to collect leaf sections onto the glass slide. To collect root sections use a 200 μL micropipettor with a cut-off tip and pipette water with root sections onto the glass slide.

**25** Check the integrity of the collected sections under a bright field microscope (using 4×, 10× and 20× objectives). If the sections look ‘blurred’ , the tissue is not cut at a perpendicular angle and the adjustable stage needs to be tilted/rotated in order to correct the orientation of the block, ensuring the long axis of the tissue is perpendicular to the blade.

**26** If the sections are intact and appear ‘in focus’ , start collecting fresh sections into 0.2 mL PCR tubes containing sterile water with 100 U RNaseOUT (prepared at step 13). Keep tubes on ice.

**For thin fragile sections:** Sections are transferred to the silane glass slide with water and RNaseOUT (prepared at step 13). Keep the glass slide on a cold, dry surface.

Critical step

Leaves: Use a soft paintbrush to collect at least 15 leaf sections into each PCR tube containing sterile water and 100 U RNaseOUT (87.5 μL). This takes about 30–45 min per tube. Take care to collect sections to the tip of the paintbrush, as it can be difficult to remove sections from the paintbrush after transfer. If sections are not separating fully from the agarose, gently tease away the agarose using a paintbrush or tweezers.

Roots: Collect root sections using a 200 μL pipette with a cut-off tip, pipette up water with root sections from buffer tray and transfer into an empty tube. Once roots settle to the bottom of the tube, remove excess water until 20 μL remains in the tube. Alternatively, remove all water and add 20 μL water to the tube. Transfer 20 μL of water with root sections into PCR tubes containing sterile water and 100 U RNaseOUT. Repeat this process four times in total to collect at least 35 root sections in a final volume of 87.5 μL.

If multiple transcripts are to be tested, the collection of sections should be done concurrently rather than consecutively to ensure sections across different *in situ* PCRs are similarly sized and are from the same region of the tissue (i.e. collect sections from the first cut into PCR tube 1, sections from the second cut into PCR tube 2, etc.). This is important as leaf and root morphology and possibly expression of genes may vary along the tissue.

Take care to frequently change the blade or the section of the blade that is cutting the tissue, because the cut from a blunt blade tends to be jagged and uneven.

If root sections do not separate from ultra-low gelling temperature agarose after cutting, transfer them into a tube with sterile water using a paintbrush. Heat at 65°C for 10 min to melt the agarose. Wash three times with sterile 65°C warm water before proceeding.

**27** Once enough sections have been collected, disassemble the ice bath/buffer tray/specimen plate from the vibratome. Cut around the bottom of the agarose block using a single-edge blade, before slicing underneath the block to lift it off the specimen plate. If there is a substantial amount of plant material left in the block, use tweezers or paintbrushes to transfer the agarose block back into the 12-well plate. Viable tissue samples can be kept at 4°C for at least three weeks.

**28** Clean the superglue from the specimen plate by carefully scraping a single-edge blade over it, taking care not to damage the specimen plate.

### DNase treatment

#### Timing ~1 h

Critical step

Take great care when pipetting solutions into and out of 0.2 mL PCR tubes or from the glass slide containing the sections. They are very easily damaged and may stick to the pipette tip. Gently pipette up and down to mix the sample and if this not possible, flick tubes using finger. Do not use a centrifuge. Sections should never be allowed to dry; prepare mastermixes for next step during the current incubation such that as soon as solution 1 is removed, solution 2 can be added.

**29** On ice, to the 0.2 mL PCR tubes containing 87.5 μL sterile water, RNaseOUT and tissue sections, add 10 μL of 10× Turbo DNA-free buffer (Ambion) and 2.5 μL of 1 U μL^−1^ DNase I (Qiagen) (see REAGENT SETUP) to give a final volume of 100 μL.

**For both non-embedded, non-sectioned samples AND thin fragile sections:** Add the above DNase reaction mixture directly onto the tissue samples on the glass slide containing water and RNaseOUT.

**30** Incubate at 37°C for 45 min in a thermocycler with either a 0.2 ml PCR tube block or a slide block.

**31** Add 3.3 μL of 0.5 M EDTA, pH 8.0 (see REAGENT SETUP) to give a final concentration of 15 mM. Heat inactivate the DNase I enzyme at 70°C for 15 min. Place on ice.

Critical step

**For non-embedded, non-sectioned samples AND thin fragile sections:** In our experience the glass slide is prone to cracking due to expansion and contraction of the metal PCR block. To avoid this we recommend removing the slides prior to the final cooling step. For instance, if the thermal cycler is set to cool down to 4°C after the 70°C EDTA incubation in the DNase step, remove the slide in the final 5 seconds of the 70°C incubation and place on a cold (4°C) flat surface. This also applies to subsequent incubations (see steps 37 and 39).

**32** Using a 200 μL pipette, carefully remove the DNase solution and discard.

**33** Wash twice for 1 min in 150–200 μL ice-cold sterile water.

**34** Using a 200 μL pipette and 10 μL pipette gently remove as much of the water as possible.

### Reverse transcription

#### Timing ~1.5 h

Critical step

For the negative controls, add all reagents except Thermoscript RT. Alternatively exclude negative controls from the RT and keep samples at 4°C in 1× PBS during steps 35–39.

**35** On ice, to each 0.2 mL PCR tube containing the sections, add 10 μL of sterile water and 2 μL of 10 mM dNTPs.

**For non-embedded, non-sectioned samples AND thin fragile sections:** Add 60.5 μL of sterile water and 10 μL of 10 mM dNTPs.

**36** Add 1 μL of 10 μM gene-specific reverse primer.

**For non-embedded, non-sectioned samples AND thin fragile sections:** Add 2.5 μL of 10 μM gene-specific reverse primer.

Critical step

Reverse primers that have previously been used in qPCR generally work well as primer in RT and in the subsequent *in situ* PCR. Alternatively, design a new reverse primer that is 5′ of the gene-specific reverse primer used in the *in situ* PCR (see “Primer design” and “RT-PCR” for more details).

**37** In a thermal cycler, incubate samples for 5 min at 65°C and hold at 4°C for at least 1 min. Place on ice.

**For non-embedded, non-sectioned samples AND thin fragile sections:** See critical note under step 31.

**38** Add 7 μL of RT reaction solution (see REAGENT SETUP, Table 3).

**For non-embedded, non-sectioned samples AND thin fragile sections:** Add 27 μL of RT reaction solution.

**39** In a thermal cycler, incubate for 1 h at 50°C, 5 min at 85°C, and then hold at 4°C.

**For non-embedded, non-sectioned samples AND thin fragile sections:** See critical note under step 31.

PAUSE POINT.

### *In situ* PCR

#### Timing 1–2.5 h

**40** Place tubes/glass slides into a rack on ice. Using a 200 μL pipette carefully remove the RT solution.

**41** Wash twice for 1 min in 150-200 μL ice-cold sterile water.

**42** Using a 200 μL and 10 μL pipette remove as much of the water as possible.

**43** On ice, to each 0.2 mL PCR tube containing the sections add 45 μL of PCR reaction solution (see REAGENT SETUP).

**For non-embedded, non-sectioned samples AND thin fragile sections:** Add 90 μL of PCR reaction solution.

**44** Add 2.5 μL of each 10 μM gene-specific forward and reverse primers to give final reaction volume of 50 μL.

**For non-embedded, non-sectioned samples AND thin fragile sections:** Add 5 μL of each 10 μM reverse and forward primers to give a final reaction volume of 100 μL.

**45** Cycling conditions will vary according to primer Tm, product size, and the abundance of the transcript of interest. A sample protocol is given below for primers designed with an annealing temperature of 55°C and a product size of approximately 200 bp.

Initial denaturation:

98°C 30”

Cycling. Repeat 20-35× (depending on results from the Test PCR):

98°C 10”

55°C 25”

72°C 5”

Final elongation:

72°C 7’

10°C hold

**For non-embedded, non-sectioned samples AND thin fragile sections**: See critical note under step 31.

### Colourimetric detection of DIG labeled PCR products

#### Timing ~3-4 h

**For non-embedded, non-sectioned samples AND thin fragile sections:** For on-slide *in situ* PCR, remove the frame-seal chamber from the slide completely using a scalpel and complete all remaining steps on the slide. During incubations, place the slides within a small and sealable plastic box to minimize evaporation.

**46** Place 0.2 mL PCR tubes into a rack on ice. Using a 10 μL pipette, carefully pipette off the rest of the PCR solution.

**47** Wash tissue sections twice for 5 min in 150–200 μL 1× PBS (see REAGENT SETUP).

**48** Using a 200 μL and 10 μL pipette remove as much of the 1× PBS as possible.

**49** Gently add 100 μL of 1× Block solution (see REAGENT SETUP) to the sections and incubate for 30 min on ice.

**50** During incubation, dilute Anti-DIG-AP antibody 1:500 in 1× Block solution (see REAGENT SETUP). Keep on ice.

**51** Using a 200 μL pipette and 10 μL pipette, carefully pipette off as much of the 1× Block solution as possible.

**52** Add 50 μL of diluted Anti-DIG-AP antibody to the tissue sections and incubate at room temperature for 1 h.

**53** Using a 200 μL pipette, remove the antibody solution.

**54** Wash tissue sections twice for 15 min at room temperature in Wash Buffer 2. Keep sections in Wash Buffer 2 after second wash.

**55** For detection move to a Bright Field microscope equipped with a camera.

**56** Label silanised microscope slides. Label should include species, gene name, whether negative or positive control, and the date.

**57** Using a glass pipette big enough to pipette leaf sections or a 200 μL pipette with cut-off tip for root sections, transfer the sections along with Wash Buffer 2 onto a silanised microscope slide. A paintbrush should be used if any sections stick to the wall of the tube.

**58** Using a 200 μL pipette, remove any excess Wash Buffer from the slide.

Critical step

Hold pipette tip perpendicularly to the slide surface and remove liquid very slowly.

**59** Add 50 μL BM purple or any substrate for the AP enzyme to each sample on the glass slides. Some substrates, such as BM purple are light sensitive. Only take an aliquot from the stock and keep in the dark and on ice. Once added to the slides, cover the slides to protect from light and let develop in the dark until a purple-blue signal appears.

**60** Initially incubate for 10 min at room temperature. Check staining under the microscope (4× and 10× objectives, without cover slide) every 15–30 min. Keep slides in the dark.

Critical step

The time of staining may vary anywhere from 10 min to over 2 h depending on species, the abundance of the transcript, and the PCR efficiency. If no signal is detected after 1 h, pipette off the AP substrate and add fresh 50 μL BM purple. Do not leave sections to develop overnight, as they will become saturated.

### Mounting and microscopy

#### Timing ~30 min

**61** Once the stain has developed and the DIG-labeled PCR products are detected, use a 200 μL pipette remove the AP substrate from the slide.

**62** Add 100 μL Wash Buffer 2 and wash three times 5 min to remove residual stain and any debris.

**63** Wash once with 100 μL sterile water. Using a 200 μL pipette, remove all the water.

**64** Using a 200 μL pipette, mount sections in 40–150 μL of 40% glycerol. The amount of glycerol will depend on the number of sections on your slide. Take care all the sections are covered but do not use an excessive amount of glycerol.

**65** Place coverslip on top and seal corners with nail varnish. If the amount of glycerol is too much, such that the coverslip floats, pipette off some glycerol then seal.

Critical step

Do not place the coverslip by sliding down from one edge (as usually practiced), as all the sections will float to one side. Simply drop the coverslip from above.

**66** Sections may be visualized under a bright field or fluorescence microscope for the next 3 weeks. Store slides at room temperature.

## Timing overview

Steps 1–6, fixation: 1 h procedure plus 2.5 h incubation (leaves) or 19 h incubation (roots).

Steps 7 + 8, washes: 1 h.

Steps 9–12, embedding: 1 h procedure plus minimum 3 h incubation or overnight.

Steps 13–28, sectioning: 3.5 h.

Steps 29–34, DNase treatment: ~1 h.

Steps 35–39, reverse transcription: ~30 min procedure plus 1 h incubation.

Steps 40–45, *in situ* PCR: up to 2.5 h.

Steps 46–60, detection of DIG labeled PCR products: up to 4 h.

Steps 61–66, mounting: 30 min.

## Troubleshooting

Advice on troubleshooting is listed in Table 5.

**Table 5:** Troubleshooting suggestions

**Step**	**Problem**	**Possible reason**	**Solution**
3	Inconsistent staining in the positive control sample (as in Figure 4A and B)	i) RNA is degraded	i) Reduce the time between harvesting plant tissue and placing it in fixative. Follow general rules for working with RNA to prevent contamination with RNases (see also start of protocol).
		ii) Over-fixation	ii) Reduce overall fixation time, but increase pressure/time of vacuum infiltration to ensure fixative penetrates.
		iii) Under-fixation (tissue may be too large for fixative to penetrate).	iii) Pieces must be kept at a maximum of 5x5 mm to ensure fixative can penetrate entire tissue. Increase pressure/time of vacuum infiltration to ensure fixative penetrates.
11, 20	Poor morphology (as in Figure 4C)	i) Long axis of sample is not perpendicular to blade on vibratome	i) Be quick when orienting the tissue pieces in the molten agarose and place the sample on ice for the agarose to solidify. Examine the first few sections cut on the vibratome and if they appear smeared, adjust the orientation of the block by either tilting the adjustable stage or removing the agarose block and making sure the side stuck on the stage is perfectly flat.
		ii) Sections have been damaged during processing	ii) Be careful not to damage the tissue sections during processing (use a paintbrush for manipulation, do not vortex or centrifuge tubes, prevent pipette tip from contacting sections during multiple rounds of pipetting, consider performing experiments on-slide).
29	Nuclear staining throughout the sample (as in Figure 4D)	Amplification of gDNA	Design primers that are split across an exon-exon junction to eliminate the possibility of amplifying gDNA. Alternatively, design primers that have 1 or more large introns between them such that reducing the elongation time during PCR only allows amplification of the smaller cDNA product.
			Increase the incubation time of the DNase treatment or change the DNase enzyme.
45, 60	Staining of sections appears very dark	Saturated staining	Reduce the number of cycles in the PCR and/or reduce the overall staining time.
60	Positive result in the negative “no RT” control or weak non-specific staining in the positive control	Background staining caused by presence of endogenous alkaline phosphatase enzymes	Add levamisole to the substrate to inhibit endogenous alkaline phosphatases.

## Results and discussion

**Figure 3:**
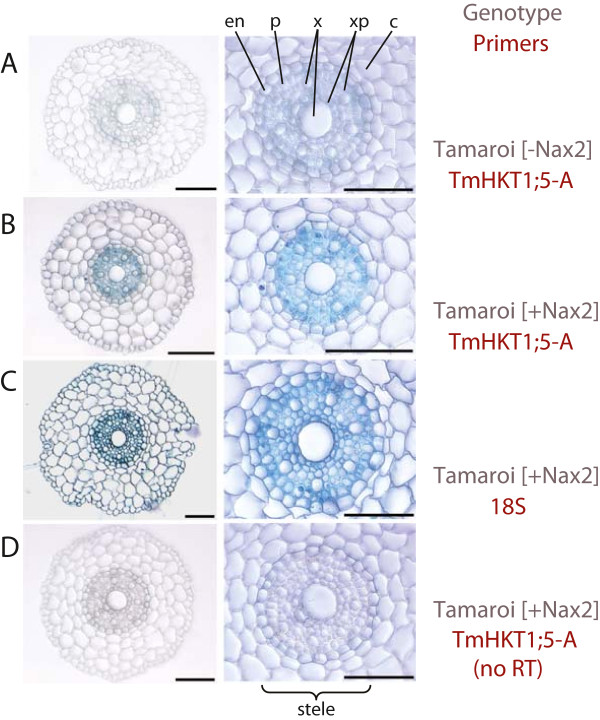
***In situ *PCR demonstrating vasculature-specific expression of a sodium transporter (*TmHKT1;5-A) *in durum wheat (*Triticum durum*) root sections.** The blue stain indicates the presence of transcripts while the images in the right panel are magnifications of figures in the left panel. **(A)** shows that *TmHKT1;5-A* is undetectable in Tamaroi [−*Nax2*] (the near-isogenic line without *Nax2/TmHKT1;5-A*). **(B)** depicts the stele-specific expression of *TmHKT1;5-A* in Tamaroi [+*Nax2*]. In **(C)** the expression of 18S ribosomal RNA is seen in all cell-types (positive control). **(D)** is the negative control where the reverse transcription (RT) step was omitted in Tamaroi [+*Nax2*]. c, cortex; en, endodermis; p, pericycle; x, xylem; xp, xylem parenchyma. Scale bars represent 100 μm. This figure was originally published in [21].

We use *in situ* PCR as a core technique for gene functional characterisation studies. All the evidence we have gathered so far suggests that it reliably localises the target transcript to the cell-types in which it is expressed. We see distinct patterns of staining when examining the localisation of different transcripts, and these are consistent across technical and biological replicates, and when available, with the results of published studies for the same gene using different transcript localization techniques. For instance, in Figure 2A, B and 3C using the primers listed in Table 6, we detect the presence of 18S ribosomal RNA in all cells, as would be expected. We have previously seen the preferential expression of *AtCAX1* in the mesophyll using single cell sampling and laser capture microdissection [6,7,30] and we show the same using *in situ* PCR (Figure 2C). Figure 3 shows the localisation of a transcript that encodes a sodium transporter in near isogenic lines (NIL) of *Triticum durum* (durum wheat) cultivar Tamaroi [21]. The near isogenic lines of durum wheat were made after introgression of a genomic fragment from *Triticum monococcum* in *Triticum durum* by conventional crossing and backcrossing with the durum wheat parent [21]. The *+ Nax2* NIL contains *Triticum monococcum TmHKT1;5-A* (Figure 3B) whereas the – Nax2 NIL lacks *TmHKT1;5-A* (Figure 3B). Homologues of this gene are expressed in the stele of bread wheat [24], rice [31], Arabidopsis [32] and other plants [33], and we see a very similar expression pattern in durum wheat (Figure 3B). Here, we also present examples of using *in situ* PCR on delicate tissues for two genes encoding putative organic acid transporters. One is present in the guard cells in epidermal peels from barley leaves (Figure 2D), a common localization for this gene family [34-36] and the other localizes to the septum and embryo of Arabidopsis flower/siliques (Figure 2E and 2F).

**Table 6:** **List of primers used during *in situ *** **PCR**

**Gene name**	**Accession number**	**Primer sequence (5′ to 3′)**
*Hv18S*	AK251731.1	**qF:** GGTAATTCCAGCTCCAAT
		**qR:** GTTTATGGTTGAGACTAG
*TmHKT1; 5-A*	DQ646339.2	**qF:** GACCACAAAAGGATAACAAGCA
		**qR:** AGAACATGACAGCAATGAGAGC
*GmPIP1; 2b*	XM_003532769.2	**qF:** TGTTTTTGTATGTGCTTGCTTG
		**qR:** TCCATTCAGAGTGTCACAAATACA
*AtCAX1*	NM_201901.3	**qF:** AGTTGCGTTAGGCTCTGC
		**qR:** TTGATGTCCCAAGTGAATG
*HvSL1251*	AK371960.1	**qF:** GGTCACAACCACGGCTATTT
		**qR:**GTCTTGAATGAGGGCAGAGC
*AtALMT14*	NM_001125913.1	**qF:** CGGTAGACATAACCCCAACG
		**qR:** TCAACACCACAATCCTGCTC

**Figure 4:**
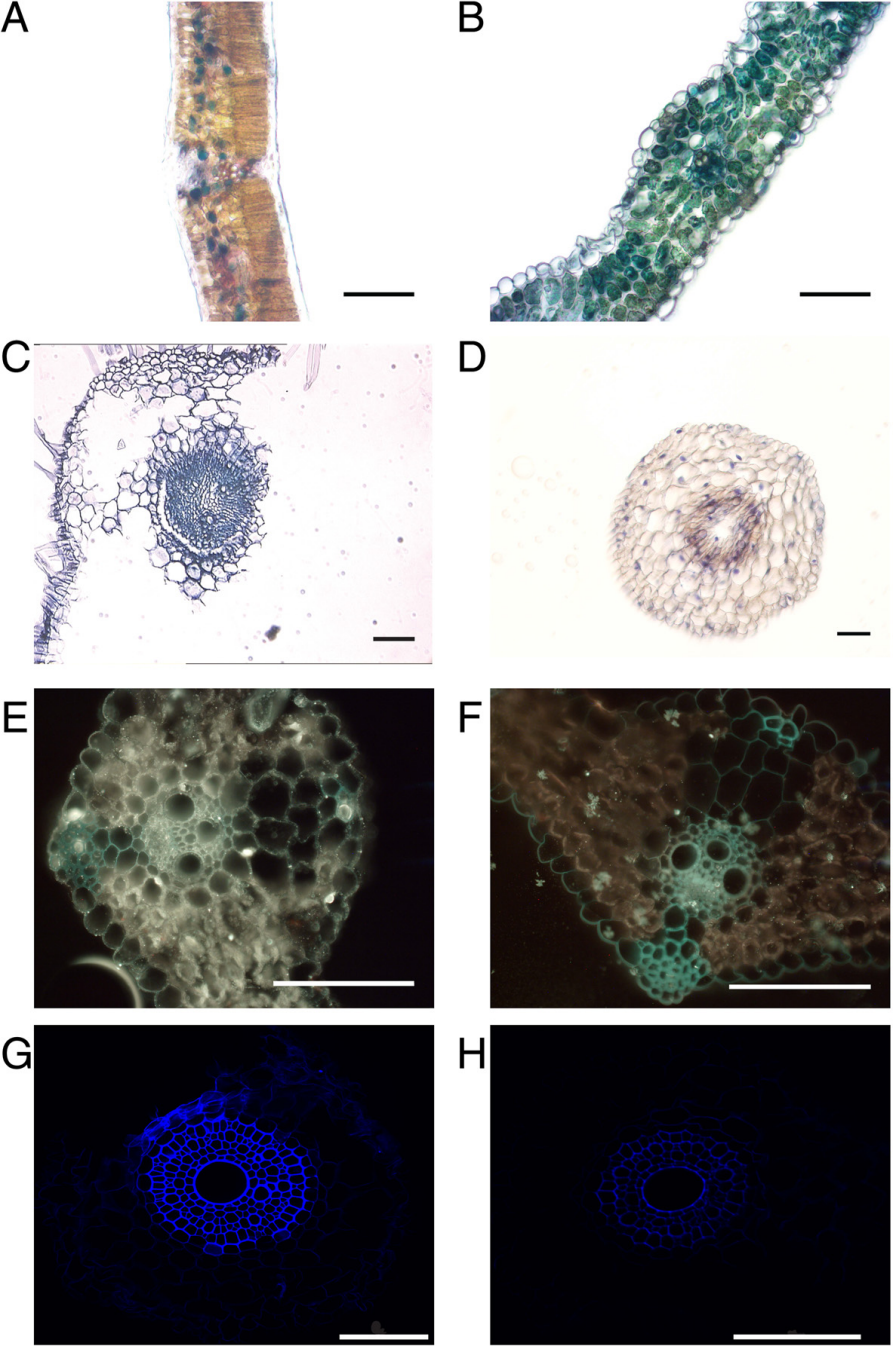
**Examples of tissue sections where *in situ *PCR was unsuccessful.** Inconsistent, non-uniform expression of 18S rRNA in *Vitis vinifera* leaf **(A)** and *Hordeum vulgare* leaf **(B)**. Severely damaged *Glycine max* root cells **(C)**. Distinct staining of cell nuclei (genomic DNA) in *Triticum durum* root section **(D)**. Fluorescent staining using Elf97 in *Hordeum vulgare* leaf (**E**, *Hv18S* rRNA and **F**, *Hv18S* rRNA negative control; Zeiss stereofluorescence microscope) and *Triticum durum* root sections (**G**, *TmHKT1; 5-A* and **H**, *TmHKT1;5-A* negative control; Nikon confocal microscope). Please see Table 5 for troubleshooting tips. Scale bars represent 100 μm.

Unfortunately, as with every PCR based technique, the experiment may sometimes fail for an unforeseen (and seemingly inexplicable reason), but, in our hands, the vast majority of experiments have yielded usable results, and if care is taken then it will consistently work. However, in the course of optimizing this technique we have encountered several examples of what may go wrong – this includes a total absence of detectible transcript, over-staining, and even erroneous detection of transcript (see also Table 5 for troubleshooting tips). Incomplete fixation of RNA and clearing of tissue can result in detection of the target transcripts in fewer cells than in which it is actually expressed. This is exemplified by the lack of detection of 18S RNA in all cell types either through poor penetration of fixative (Figure 4A) or poor clearing of chlorophyll (Figure 4B). This is the reason why it is essential to detect a ‘house-keeping’ transcript, which is equally expressed in all cell-types, at the same time as your target transcript to make sure you have complete and equal fixation and clearing. Performing sectioning on very delicate tissues, including older root tissues, is difficult as they can often fall apart during processing (Figure 4C) so these types of samples are best processed on slides. Figure 4D shows an example of what can happen if the primers detect genomic DNA; the nucleus stains and shows a pattern quite distinctive from cytoplasmic cDNA staining. This scenario can be avoided by optimal primer design.

We attempted to use fluorescence detection of gene expression in tissue sections as we hypothesized we would achieve an improvement over what is possible with colourimetric detection in terms of signal intensity and contrast with the background image. However, due to the inherent autofluorescence in our no RT control sections (of chlorophyll in leaf tissue, and vascular and cuticular regions in leaf and root tissue control samples (Figure 4E–H)) the use of fluorescence was less satisfactory as a detection method for gene expression in our hands. For instance, when we used fluorescence to investigate the expression of *TmHKT1;5-A* in the *+ Nax2* NIL we detected a similar signal in our no RT negative control and our sample (Figure 4G and H); this contrasts with no signal in our no RT control using colourimetric detection (Figure 3D) or in the NIL *–Nax2* (Figure 3A). Despite the lower signal of the autofluorescence we could not eliminate it using lower gain or spectral unmixing when using confocal microscopy. The use of fluorescence also has additional complications compared to the more reliable and straightforward colourimetric detection (such as cost of fluorescent substrates or primers, stability of fluorescent compounds and the cost and access to fluorescence/confocal microscopes). Pesquet et al. [18] used fluorescence to detect *in situ* PCR products in plant tissues, however the protocol was not outlined and the technique has not been used very often; problems with autofluorescence interference when using fluorescent detection of *in situ* PCR products have since been noted [37].

## Conclusion

We advocate the use of colourimetric detection of *in situ* PCR products for gene expression localization in plant tissue. Once the primers have been validated to be specific to the required target, this technique is a relatively quick and powerful tool to spatially define the expression profile of any gene of interest. We recommend the adoption of this technique as a standard method for the localization of genes in plant tissue.

## Competing interests

The authors declare that they have no competing interest.

## Authors’ contributions

MG, SKT and AA wrote the manuscript. RAB and SJC initiated the development of the *in situ* technique on plant tissue, GMM provided advice and training on microtomy, AA conducted most of the development (with CJ) and performed the wheat and soybean *in situ* PCR, CJ performed Arabidopsis CAX experiments, VMC performed and developed the technique for on-slide PCR, and performed the experiments on barley, epidermal peels and flowers. WWN performed additional experiments on barley. All authors commented on the manuscript.
